# Non-coding RNAs: Key players in T cell exhaustion

**DOI:** 10.3389/fimmu.2022.959729

**Published:** 2022-10-04

**Authors:** Kun Li, Ziqiang Wang

**Affiliations:** ^1^ Department of Nuclear Medicine, The First Affiliated Hospital of Shandong First Medical University & Shandong Provincial Qianfoshan Hospital, Jinan, China; ^2^ Biomedical Sciences College & Shandong Medicinal Biotechnology Centre, Shandong First Medical University & Shandong Academy of Medical Sciences, Jinan, China

**Keywords:** non-coding RNA, miRNA, lncRNA, T cell exhaustion, immune checkpoint, chronic viral infections, tumorigenesis

## Abstract

T cell exhaustion caused by continuous antigen stimulation in chronic viral infections and the tumor microenvironment is a major barrier to successful elimination of viruses and tumor cells. Although immune checkpoint inhibitors should reverse T cell exhaustion, shortcomings, such as off-target effects and single targets, limit their application. Therefore, it is important to identify molecular targets in effector T cells that simultaneously regulate the expression of multiple immune checkpoints. Over the past few years, non-coding RNAs, including microRNAs and long non-coding RNAs, have been shown to participate in the immune response against viral infections and tumors. In this review, we focus on the roles and underlying mechanisms of microRNAs and long non-coding RNAs in the regulation of T cell exhaustion during chronic viral infections and tumorigenesis. We hope that this review will stimulate research to provide more precise and effective immunotherapies against viral infections and tumors.

## Introduction

T cell exhaustion was first observed in murine models of chronic lymphocytic choriomeningitis virus (LCMV) infection. Researchers have found that virus-specific CD8+ T cells can persist indefinitely in chronically infected hosts but are unable to develop antiviral effector functions and thus fail to control viral infection (1, 2). Since then, several types of viruses have been shown to induce T cell exhaustion during viral infections, including herpes simplex virus type 1 (HSV-1), human immunodeficiency virus (HIV), hepatitis B virus (HBV), hepatitis C virus (HCV), Epstein–Barr virus, and severe acute respiratory syndrome coronavirus 2 (SARS-CoV-2), a recently identified coronavirus that continuously affects millions of people (3). Growing evidence has revealed that exhausted T cells are also widely distributed in the tumor microenvironment because of persistent exposure to tumor antigens (4–6). During chronic viral infections and tumorigenesis, the exhaustion of virus- or tumor-specific CD4+ and CD8+ T cells is characterized by elevated levels of immune checkpoints, such as programmed death-1 (PD-1), programmed death ligand-1 (PD-L1), cytotoxic T-lymphocyte antigen 4 (CTLA4), T-cell immunoglobulin domain and mucin domain 3 (TIM-3), B- and T-lymphocyte attenuator (BTLA), lymphocyte activation gene 3 (LAG3), and cluster of differentiation 244 (CD244, also known as 2B4). These cells have decreased ability to produce proinflammatory cytokines, such as interleukin-2 (IL-2), tumor necrosis factor-α (TNF-α), and interferon-γ (IFN-γ), and exhibit impaired T cell proliferation (7).

**Figure 1:**
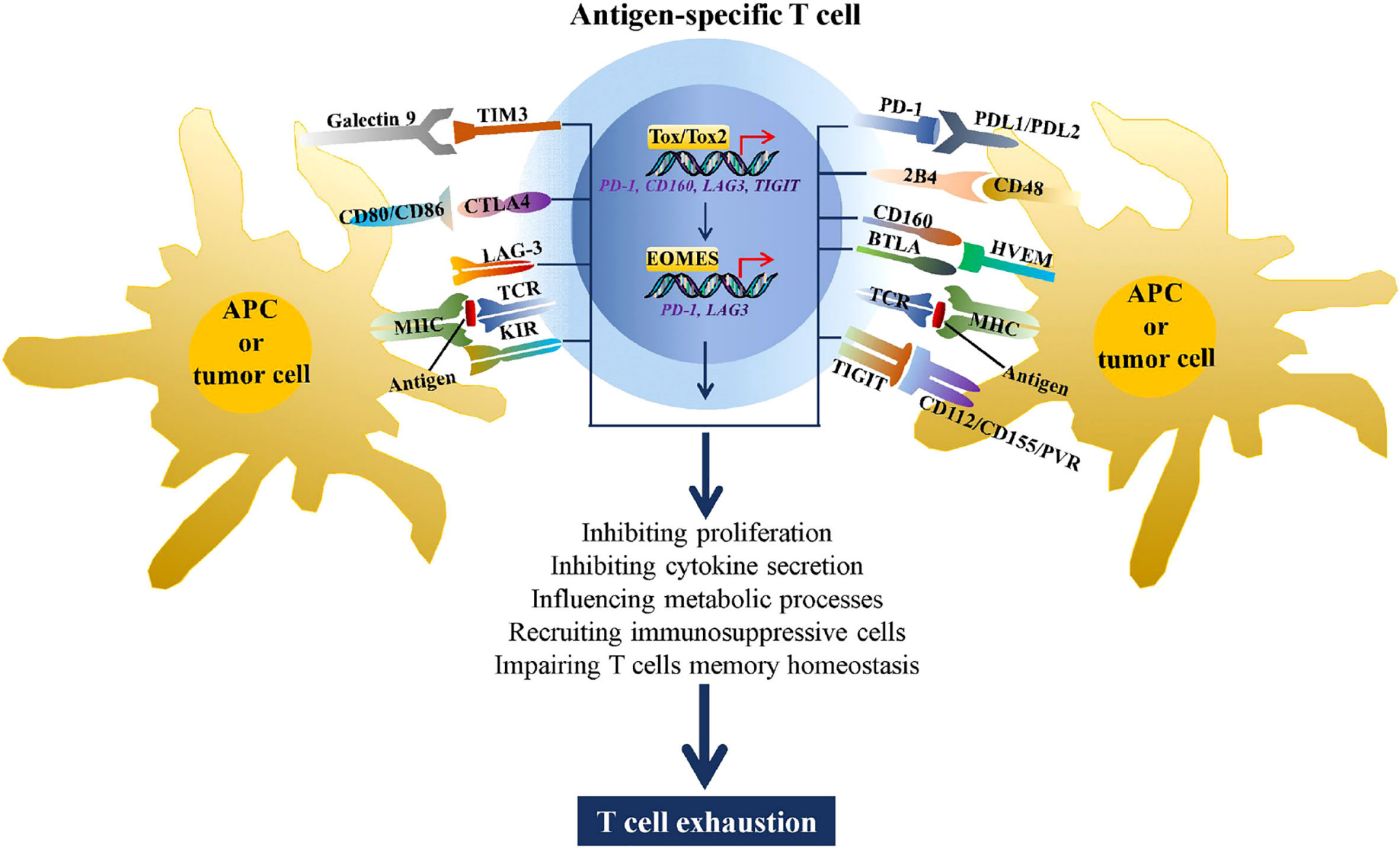
Immune checkpoints increase T cell exhaustion. During chronic viral infections and tumorigenesis, CD4+ and CD8+ T cells recognize antigens presented by MHC complexes on antigen-presenting cells (APCs) or tumor cells through the TCR, stimulating expression of multiple immune checkpoints, such as TIM3, CTLA4 LAG3, killer cell immunoglobulin-like receptor (KIR), PD-1, 2B4, CD160, BTLA, and TIGIT. By binding to the corresponding ligands, these immune checkpoints can drive T cell exhaustion through metabolic processes, inhibiting proliferation and cytokine secretion, recruiting immunosuppressive cells, and impairing T cell memory homeostasis. In addition, the high antigen stimulation of TCR induced expression of TOX/TOX2 and EOMES, two transcriptional factors that are involved in promoting T cell exhaustion by regulating transcription of PD-1, CD160, LAG3, and TIGIT (13–15).

A number of studies have demonstrated that abnormally high expression of immune checkpoints is a hallmark of T cell exhaustion (8–10). By binding to the corresponding ligands, immune checkpoints can induce T cell dysfunction through a variety of mechanisms, including metabolic processes, proliferation, cytokine secretion, recruiting immunosuppressive cells, and impairing T cell memory homeostasis (11, 12) (**Figure 1**). Although many studies have attempted to reverse T cell exhaustion using immune checkpoint inhibitors, the primary or secondary treatment tolerance, general toxicity, and immune-related adverse events observed in most individuals who receive immune checkpoint blockade therapy. These effects significantly limit their clinical use because of their shortcomings, such as off-target effects and single targets (16). Thus, understanding the regulatory mechanism underlying the expression of these immune checkpoints and identifying molecular targets in effector T cells that can simultaneously regulate the expression of multiple immune checkpoints warrant further research.

Non-coding RNAs (ncRNAs), including microRNAs (miRNAs) and long ncRNAs (lncRNAs), are RNA molecules that are not translated into protein products. miRNAs are a group of evolutionarily conserved ncRNAs ranging from 19 to 24 nucleotides in length that play significant roles in post-transcriptional gene regulation through mRNA degradation or translational repression (17). lncRNAs are long functional ncRNAs that are more than 200 nucleotides long (18). Several biological processes are known to be controlled by miRNAs and lncRNAs, including cell differentiation, proliferation, ossification, autophagy, and apoptosis (19–24). miRNAs and lncRNAs also play critical regulatory roles in the progression of various disorders, such as cancers, viral infections, and neurodegenerative diseases (25–30). There is growing evidence that miRNAs and lncRNAs act as regulators of immune escape by directly or indirectly modulating the expression of immune-regulating molecules, especially immune checkpoint proteins, such as members of the CD28, B7, TNF, and TNF receptor families (31, 32). Additionally, miRNAs and lncRNAs play important roles in the differentiation, proliferation, and cytokine secretion of CD4+ and CD8+ T cells (33). Many miRNAs and lncRNAs have been demonstrated to be dysregulated following continuous exposure to antigens in viral infections and tumors, and these miRNAs and lncRNAs play crucial roles in the maintenance of T cell functions (34–37), suggesting that miRNAs and lncRNAs have great potential as therapeutic targets for reversing T cell exhaustion.

In this review, we provide an overview of the roles of miRNAs and lncRNAs in T cell exhaustion during chronic viral infections and tumorigenesis and present their potential implications in reversing T cell exhaustion against human cancers and viral infections, with an emphasis on immune checkpoint regulation. We hope that this review will contribute to the development of non-coding RNA-based therapeutic approaches for the treatment of any type of RNA-related disease, such as SARS-CoV-2.

## Chronic viral infections

An increasing number of studies have confirmed that the antigenic load during viral infections directly contributes to CD4+ and CD8+ T cell exhaustion, and several ncRNAs have been identified to be involved. In this section, we summarize the roles of ncRNAs in T cell exhaustion during chronic HSV-1, LCMV, and HIV infections (**Table 1**).

**Table 1:** Roles of of ncRNAs in T cell exhaustion during chronic viral infections.

NcRNA	Disease	Target	Role	Reference
LAT	HSV-1 infection	PD-1, TIM-3	Promoting CD8+ T cells exhaustion	(38, 39)
		PD-L1	Promoting CD8+ T cells exhaustion	(40)
		PD-1, TIM-3, CTLA-4	Promoting CD8+ T cells exhaustion	(41)
miR-31	LCMV infection	c-Maf, Ptger2	Promoting CD8+ T cells exhaustion	(42)
miR-155		PD-1, CD160, 2B4	Promoting CD8+ T cells exhaustion	(43)
miR-9	HIV-1 infection	Blimp-1	Inhibiting CD4+ T cells exhaustion	(44, 45)
let-7		IL-10	Inhibiting CD4+ T cells exhaustion	(46)
miR-146a		PD-1, CTLA-4	Promoting CD4+ T cells exhaustion	(47, 48)

## Herpes simplex virus type 1

HSV-1 is a human alpha herpesvirus closely related to orofacial, genital herpes, and encephalitis (49). HSV-1 infection is quite prevalent, with 80–90% of the population infected with HSV-1 during their lifetime. After infection, HSV-1 hijacks host cell factors, including miRNAs and lncRNAs, to facilitate viral replication and gene expression (50–53). A growing body of evidence has revealed that the HSV-1 latency-associated transcript (LAT), a viral lncRNA that is abundantly expressed during latent infection and plays a vital role in the HSV-1 latency reactivation cycle, is an important mediator that promotes viral survival and immune evasion by increasing immune checkpoint expression, thus inducing exhaustion of HSV-1 specific T cells. For example, Homayon et al. found that increased latency in the mouse trigeminal ganglia is associated with higher levels of the exhaustion markers PD-1 and TIM-3, suggesting the functional significance of T cell exhaustion in HSV-1 establishing latency. Further investigation revealed that LAT+ viruses contain more CD8+ T cells expressing PD-1 and TIM-3 than those in LAT- viruses and that CD8α+ dendritic cells are involved in LAT-induced expression of PD-1 and TIM-3 (29, 38, 54). In addition, Lbachir et al. reported that HSV-1 LAT inhibited the cytotoxic function of HSV-1-specific CD8+ T cells and decreased IFN-γ and TNF-α production by directly or indirectly upregulating PD-L1 expression in mouse neuroblastoma cells (40). Moreover, they found that LAT interferes with the phenotypic and functional maturation of HSV-1 antigen-positive dendritic cells and upregulates the expression of PD-1, TIM-3, and CTLA-4 in HSV-specific CD8+ T cells (41, 55).

## Lymphocytic choriomeningitis virus

LCMV infection in mice is a well-established model for investigating the mechanism underlying T cell exhaustion induced by chronic viral infection (56). In studies investigating the role and molecular mechanism of miRNAs during T cell activation and differentiation, miR-31 expression was found to be strongly enhanced by activation of the T cell antigen receptor (TCR) in a calcium- and NFAT-dependent manner (42, 57, 58). Further studies confirmed that miR-31 promotes CD8+ T cell exhaustion and inhibits T cell responses to LCMV infection by increasing expression of c-Maf, a transcription factor, and Ptger2, the receptor for prostaglandin E2 (Ptger2), which contributes to CD8+ T cell dysfunction by upregulating several immune checkpoints, such as PD-1, LAG3, and IL-10 (59–62), and downregulating the expression of several T cell effector molecules, including perforin, granzymes, and osteopontin (42).

miR-155 is another miRNA, the expression of which is affected by TCR activation. Romero et al. reported that miR-155 functions as a key molecule for effector CD8+ T cell accumulation and response to LCMV infection by targeting suppressor of cytokine signaling (SOCS-1), thus promoting cytokine responsiveness and accumulation (63). However, Wherry et al. found increased miR-155 expression in exhausted T cells during LCMV infection and that miR-155 promotes the development, proliferation, accumulation, and long-term durability of terminally differentiated and exhausted T cells through the activator protein 1 (AP-1) transcription factor pathway. Moreover, miR-155 reduced the percentage of CD8+ T cells producing IFN-γ and TNF-α and promoted T cell exhaustion by upregulating the expression of immune checkpoints, including PD-1, CD160, and 2B4 (43).

## Human immunodeficiency virus

Functional exhaustion of immune cells is also a defining characteristic of HIV-1 chronic infection, and several miRNAs have been identified to be involved in the development of T cell exhaustion observed in HIV-1 infection, including miR-9, miRNA let-7, and miR-146a. Kelleher et al. found that downregulation of miR-9 in progressive HIV-1 infection upregulated the expression of B lymphocyte-induced maturation protein 1 (Blimp-1), a transcriptional repressor of IL-2 expression, and was correlated with PD-1, LAG3, 2B4, IFN-γ, and TNF-α (44, 45, 64). miRNA let-7 is another miRNA that was shown to be significantly downregulated in CD4+ T cells from HIV-1 infected patients, and miRNA let-7 depletion increased the expression of IL-10, an immunosuppressive cytokine that drives the exhaustion of intratumoral CD8+ T cells through a pathway dependent on the transcription factor Blimp-1 (45, 65, 66). miR-146a expression is induced during acute and chronic HIV-1 infections (67, 68). Feng et al. found that chronic HIV-1 infection upregulated the expression of miR-146a and exhaustion markers PD-1 and CTLA-4 following T cell activation, and the depletion of miR-146a improved the antiviral capacity of peripheral blood mononuclear cells from chronic HIV-1 infected patients through the upregulation of antiviral cytokines, such as IFN-γ, IL-2, and TNF-α. Further investigation revealed that miR-146a positively regulates the expression of PD-1 and CTLA-4 (47, 48). These findings suggest that the miR-9/Blimp-1, let-7/IL-10, and miR-146a/PD-1/CTLA-4 axes play important roles in CD4+ T cell dysfunction observed in HIV-1 infection.

## Tumorigenesis

In the tumor microenvironment, tumor cells can negatively regulate the functions of tumor-infiltrating T lymphocytes through their association with immunosuppressive cells and the secretion of immunosuppressive cytokines that inhibit tumor-infiltrating T lymphocytes activation by stimulating the expression of multiple inhibitor receptors through binding to cytokine receptors and then triggering signal transducer and activator of transcription (STAT)-dependent signaling pathway (69). In this section, we summarized the roles of ncRNAs in T cell exhaustion during tumorigenesis of melanoma, hepatocellular carcinoma (HCC), breast cancer, nasopharyngeal carcinoma (NPC), gliomas, and lung adenocarcinoma (**Table 2**).

**Table 2:** Roles of ncRNAs in T cell exhaustion during tumorigenesis.

NcRNA	Disease	Target	Role	Reference
miR-28	Melanoma	PD-1, TIM-3, BTLA	Inhibiting exhaustion of CD4+, CD8+ T cells and Treg cells	(70)
miR-155		SOCS-1	Inhibiting CD8+ T cells exhaustion	(63)
		Ship1	Inhibiting CD8+ T cells exhaustion	(71)
miR-200c	HCC	PD-L1	Inhibiting CD8+ T cells exhaustion	(72)
lnc-Tim3		TIM-3	Promoting CD8+ T cells exhaustion	(73)
miR-149-3p	Breast cancer	PD-1, TIM-3, BTLA, Foxp1	Inhibiting CD8+ T cells exhaustion	(74)
miR-424-5p		PD-L1	Inhibiting CD8+ T cells exhaustion	(75)
miRNA-138-5p		PD-L1	Inhibiting CD8+ T cells exhaustion	(76)
miR-24	NPC	CD39, PD-1, TIM-3	Promoting CD4+ T cells exhaustion	(77)
miR-15a/16	Glioma	PD-1, TIM-3, LAG-3	Promoting CD8+ T cells exhaustion	(78)
circRNA-002178	Lung adenocarcinoma	PD-L1, PD-1	Promoting CD8+ T cells exhaustion	(79)

## Melanoma

Melanoma, the most aggressive type of skin cancer, is a common cancer in the western world, increasing in incidence (80), although a rapid decline in death rates has been reported in the United States since the introduction of immune checkpoint inhibitors (81). In a study of the underlying roles and mechanisms of miR-155 in melanoma progression, miR-155 expression was found to be upregulated in effector CD8+ T cells depending on the strength of TCR stimulation and differentiation, and miR-155 knockout promoted tumor growth by enhancing the SOCS-1/STAT5 signaling pathway, thereby impairing cytokine production and resulting in CD8+ T cell exhaustion (63). In addition, miR-155 was found to inhibit T cell senescence and exhaustion to enhance the antitumor response by silencing key transcriptional factors that drive terminal differentiation and exhaustion of T cells *via* the Ship1/Phf19/Polycomb repressor complex 2 (PRC2) axis (71). Moreover, Li et al. reported that miR-28 was significantly altered in exhausted T cells isolated from a murine melanoma model. Further investigation revealed that miR-28 is involved in T cell exhaustion by directly regulating several immune checkpoints, including PD-1, TIM-3, and BTLA (70).

## Hepatocellular carcinoma

HCC was the seventh most common cancer and the second leading cause of cancer-related deaths worldwide in 2020 (80). The main risk factors for HCC are chronic HBV or hepatitis C viral infections (82). A study investigating the regulatory network between host factors and T cell exhaustion in HBV-related hepatocellular carcinoma revealed an HBV/pSTAT3/SALL4/miR-200c axis regulated PD-L1 expression and confirmed that miR-200c functions as a therapeutic target to reverse T cell exhaustion during HCC progression (72). In addition, lnc-Tim3 can induce the exhaustion of CD8+ T cells by specifically binding to TIM-3 to upregulate the expression of anti-apoptotic proteins by releasing Bat3 and promoting the activation of p53 and RELA (73).

## Breast cancer

Breast cancer remains the most diagnosed cancer and the leading cause of cancer-related deaths in women, with an estimated 2.3 million new cases worldwide by 2022 (80). Zhang et al. studied the role of miRNAs in human breast cancer progression and found that the expression of 117 miRNAs was significantly altered in CD8+ PD-1+ and CD8+ PD-1– T cells isolated from 4T1 breast tumor-bearing mice. Among them, miR-149-3p has been shown to promote T cell proliferation, secretion of cytokines IL-2, TNF-α, and IFN-γ, and cytotoxicity of CD8+ T cells against 4T1 breast tumor cells. Further investigation of the molecular mechanism demonstrated that upregulation of miR-149-3p reversed T cell exhaustion by targeting several immune checkpoints, including PD-1, TIM-3, BTLA, and Foxp1 (74). In addition, two downregulated miRNAs, miR-424-5p (75) and miR-138-5p (76), were shown to reduce breast cancer cell viability and restrain T cell exhaustion by upregulating the expression of the effector cytokines IL-2, TNF-α, and IFN-γ, and downregulating the expression of the regulatory cytokine IL-10 by targeting PD-L1.

## Nasopharyngeal carcinoma

NPC is a common head and neck cancer of nasopharyngeal epithelium (80). A study to understand the mechanism of T cell development in the tumor microenvironment of NPC found that hypoxia-induced overexpression of miR-24 promoted the transition of activated T cells to exhausted T cells by increasing the expression of CD39, PD-1, and TIM-3. Further investigation revealed that miR-24 reduced ATP production and decreased mitochondrial mass in T cells by binding to and repressing the expression of the proto-oncogene Myc and fibroblast growth factor 11, two essential factors for the reprogramming of mitochondrial dynamics (77).

## Gliomas

Gliomas are the most common and aggressive tumors that arise in the central nervous system and are characterized by poor prognosis (83). Researchers have studied the effects of miR-15a/16 on the antitumor immune response in glioma-infiltrating CD8+ T cells and observed that an miR-15a/16 deficiency in an orthotropic GL261 mouse glioma model attenuated cancer progression by inhibiting tumor growth and prolonged mouse survival. Mechanistic investigations revealed that miR-15a/16 deficiency resulted in tumor-infiltrating CD8+ T cells accumulation and promoted the secretion of IFN-γ, IL-2, and TNF-α. Moreover, miR-15a/16 knockout resulted in lower expression of PD-1, TIM-3, and LAG-3 by targeting mTOR, a crucial regulator of T cell functions (78, 84).

## Lung adenocarcinoma

Lung cancer remains the leading cause of cancer-related deaths, with an estimated 2.2 million new cases and 1.8 million deaths worldwide in 2020 (80). Lung adenocarcinoma usually originates from the mucosal glands and represents approximately 40% of all lung cancers. In a study that determined the function and mechanism of circular RNAs (circRNAs) in lung adenocarcinoma, has-circRNA-002178 expression was significantly upregulated in lung adenocarcinoma cells and tissues. This high expression of has-circRNA-002178 induced T cell exhaustion by sponging miR-34 to enhance PD-L1 expression in cancer cells. Moreover, cancer cell-derived circRNA-002178 can be delivered into CD8+ T cells *via* exosomes, resulting in increased PD-1 expression (79).

## Discussion

**Figure 2:**
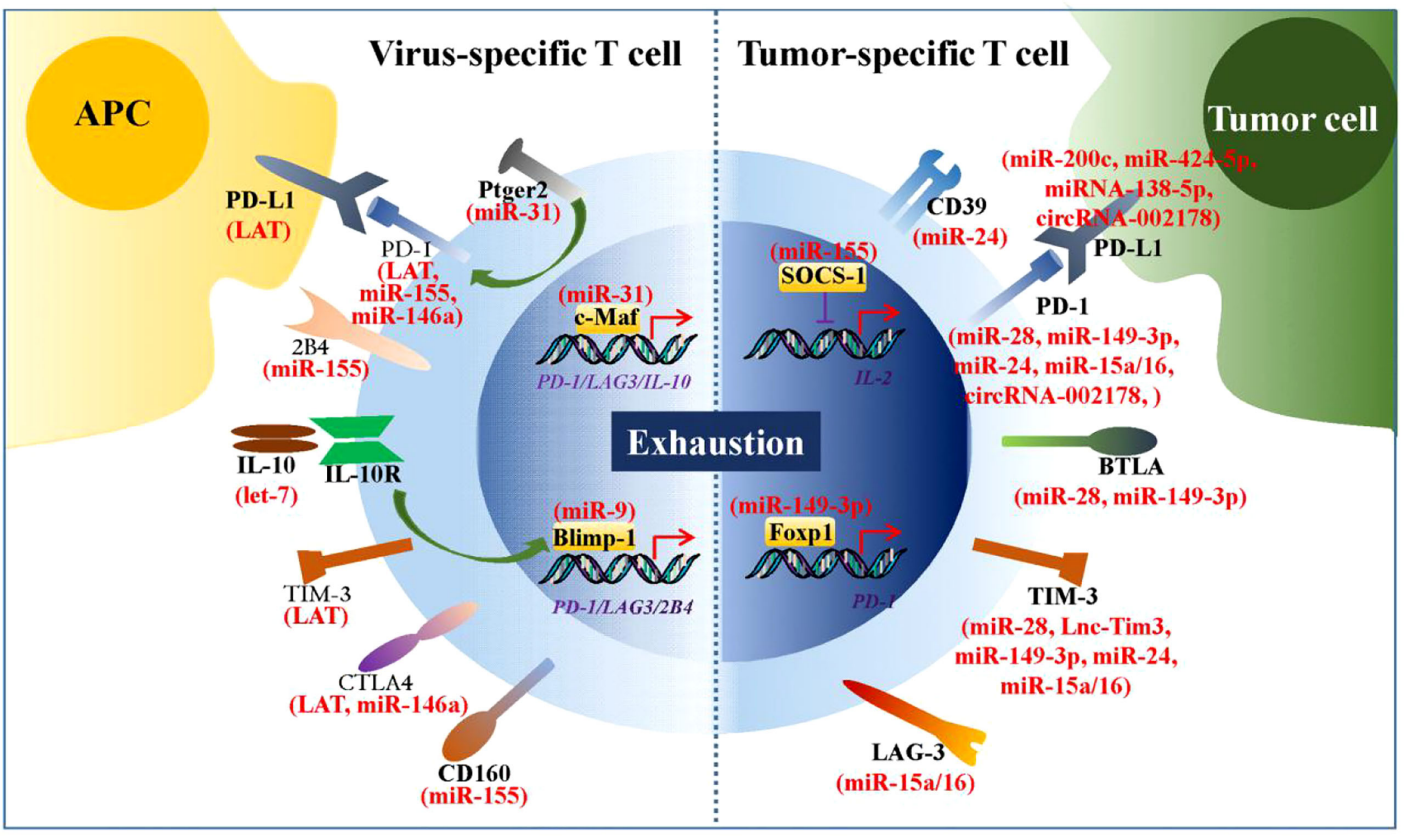
ncRNAs regulate expression of numerous immune checkpoints. During chronic viral infections and tumorigenesis, numbers of ncRNAs are found to be involved in virus- and tumor cell-induced T cell exhaustion by regulating expression of immune checkpoints. These immune checkpoints are mainly inhibitory receptors or the transcriptional factors that regulate their transcription.

Viral infections and tumors are two classes of diseases that pose significant threats to humans (80, 85). During viral infections and tumorigenesis, effector CD4+ and CD8+ T cells act as master players of the adaptive immune system to eradicate the virus and tumor cells from the host. However, T cells chronically exposed to viral and tumor antigens gradually lose their ability to control viral replication and tumor cell growth (86). Thus, T cell-based immunotherapy is a promising approach for the treatment of viral infections and tumors. A growing body of evidence suggests that the ncRNA-mediated upregulation of numerous immune checkpoints is involved in virus- and tumor cell-induced T cell exhaustion (**Figure 2**). Therefore, ncRNAs are of relevant research interest, and their roles in reversing T cell exhaustion should be considered.

To date, 11 RNA-based therapeutics, including small interfering RNAs (siRNAs) and antisense oligonucleotides (ASOs), have been approved by the Food and Drug Administration (FDA) and the European Medicines Agency (EMA) for the treatment of liver, muscle, or central nervous system-related diseases (87). In addition, several miRNA-based therapeutics are in phase II or III clinical development for the treatment of keloids, HCV infection, type II diabetes, nonalcoholic fatty liver disease, and Huntington’s disease, but no lncRNA-based therapeutic approaches have entered clinical development. Key challenges facing miRNA- and lncRNA-based therapeutics are the hurdles of low specificity, specific delivery, and immune responses. Therefore, technical advancements in molecular biology, immunology, pharmacology, chemistry, and nanotechnology are needed to improve specificity, tolerance, and delivery.

In addition, it should be investigated how T cell exhaustion-related ncRNAs develop altered expression, along with whether these ncRNAs interact and coordinate to regulate target genes, especially since they are powerful regulators of gene expression. Further studies are needed to elucidate the role of ncRNAs in the immune microenvironment, such as function of ncRNAs as “messenger RNAs” for communication between T cells and tumor/virus-infected cells. More importantly, several groups have reported that the number of T cell exhaustion markers was increased in CD4+ and CD8+ T cells in patients suffering from SARS-CoV-2 infection (88–90). Thus, more attention should be directed towards how to reverse T cell exhaustion during SARS-CoV-2 infection and whether ncRNAs can act as useful therapeutic targets for the treatment.

Overall, this review summarizes and discusses the roles of ncRNAs in T cell exhaustion during chronic viral infections and tumorigenesis and indicates that ncRNAs may be potent targets for enhancing T cell-based immunotherapy against chronic viral infections and tumors.

## Author contributions

KL and ZW prepared the manuscript. ZW reviewed and edited the manuscript. All authors contributed to the article and approved the submitted version.

## Funding

This work was supported by the National Natural Science Foundation of China (32000878), Shandong Provincial Natural Science Foundation (ZR2020LZL008, ZR2021LSW017), and the Academic Promotion Programme of Shandong First Medical University (2019LJ001).

## Conflict of interest

The authors declare that the research was conducted in the absence of any commercial or financial relationships that could be construed as a potential conflict of interest.

## Publisher’s note

All claims expressed in this article are solely those of the authors and do not necessarily represent those of their affiliated organizations, or those of the publisher, the editors and the reviewers. Any product that may be evaluated in this article, or claim that may be made by its manufacturer, is not guaranteed or endorsed by the publisher.

## Abbreviations

LCMV, Lymphocytic choriomeningitis virus; HSV-1, Herpes simplex virus type 1; HIV, Human immunodeficiency virus; HBV, Hepatitis B virus; SARS-CoV-2, Severe acute respiratory syndrome coronavirus 2; PD-1, Programmed death-1; PD-L1, Programmed death ligand-1; CTLA4, Cytotoxic T-lymphocyte antigen 4; TIM-3, T-cell immunoglobulin domain and mucin domain 3; BTLA, B- and T-lymphocyte attenuator; LAG3, Lymphocyte activation gene 3; CD244, Cluster of differentiation 244; IL-2, Interlekin-2; TNF-α, Tumor necrosis factor-α; IFN-γ, Interferon-γ; NcRNAs, Non-coding RNAs; MiRNAs, MicroRNAs; LncRNAs, Long non-coding RNAs; LAT, Latency-associated transcript; TCR, T cell antigen receptor; Ptger2, Prostaglandin E2; SOCS-1, Suppressor of cytokine signaling; Blimp-1, B lymphocyte-induced maturation protein 1; HCC, Hepatocellular carcinoma; NPC, Nasopharyngeal carcinoma; PRC2, Polycomb repressor complex 2; CircRNAs, Circular RNAs.
